# Nail structural alterations and zinc levels in the elderly: an observational cross-sectional study

**DOI:** 10.7717/peerj.20771

**Published:** 2026-02-03

**Authors:** Shannaz Nadia Yusharyahya, Chia-Yu Chu, Roro Inge Ade Krisanti, Lili Legiawati, Rinadewi Astriningrum, Levina Ameline Moelyono, Viecky M.P. Betavani, Valdi Ven Japranata

**Affiliations:** 1 Department of Dermatology and Venereology, Faculty of Medicine, Universitas Indonesia - Dr. Cipto Mangunkusumo Hospital, Jakarta Pusat, Indonesia; 2 Department of Dermatology, National Taiwan University Hospital, Taipei, Taiwan; 3 Faculty of Medicine, Universitas Indonesia, Jakarta Pusat, Indonesia; 4 Harvard Medical School, Boston, MA, United States of America

**Keywords:** Aging, Dermoscopy, Nail changes, Older adults, Zinc

## Abstract

**Background:**

Nails in the elderly undergo several structural changes related to aging with respect to surface, thickness, color, and growth pattern. The present study explores the potential association between nail alterations and zinc levels in this population.

**Methods:**

A total of 64 subjects aged ≥60 years with or without nail changes were recruited from the Dermatology and Venereology Outpatient Clinic at Dr. Cipto Mangunkusumo Hospital, Jakarta, Indonesia. Their nail features were observed clinically and evaluated utilizing dermoscopy, and nail clippings weighing a total of 200 milligrams were collected from each subject for nail zinc concentration measurement. Appropriate statistical tests were employed to determine the relationship between nail zinc levels and the structural alterations, as well as the patients’ comorbidities and medications, at a significance level of 0.05.

**Results:**

Most subjects in our study showed altered nail profiles in multiple digits (75.0%) with a predilection for toenails (62.3%), and the first toenail was primarily affected. The commonest dermoscopic features displayed in altered nails include nail plate pigmentation (*n* = 64), distal edge thickening (*n* = 39), and surface changes (*n* = 34). The average nail zinc concentration was lower in the elderly with nail changes than in those without, albeit with no statistically significant differences (*p* = 0.687). Subgroup analyses according to comorbidities (hypertension, diabetes mellitus type 2, and dyslipidemia) and medications (antihypertensives, antidiabetics, antidyslipidemic drugs, and anticonvulsants) also exhibited no discrepancies.

**Conclusions:**

This study highlights the complex interactions among nail structural changes, zinc levels, comorbidities, and medications; hence, further research is warranted to obtain a deeper understanding.

## Introduction

Nail changes represent a diverse group of both physiological and pathological conditions involving various nail anatomical parts, including the nail bed, nail folds, nail matrix, hyponychium, and nail plate. These alterations may manifest in regard to the nail surface texture, thickness, color, growth rate, contour, and biochemical compositions. The etiology of these conditions is typically multifactorial and may be linked with skin and systemic disorders. The natural aging process in older adults influences nail integrity due to diminished peripheral circulation and histological changes of connective and elastic tissues in the nail bed (Albucker, Conway & Lipner, 2024; Rubeiz, Abbas & Kibbi, 2010). Additionally, given the increased prevalence of underlying diseases in the elderly population, they are subject to polypharmacy, and multiple drug use possibly interacts and decreases the efficacy of medications for treating nail disorders (Abdullah & Abbas, 2011; Albucker, Conway & Lipner, 2024), or interferes with the absorption and bioavailability of nutrients that constitute nail structures (Prescott, Drake & Stevens, 2018). Other factors predisposing the elderly population to nail changes are the heightened susceptibility to infection, trauma, and disturbances in nail biomechanics (El-Domyati, Abdel-Wahab & Abdel-Azim, 2014).

All of these factors, whether solely or in combination, contribute to the pervasive nail alterations in older adults. Sarshar et al. (2022) reported that about 95.5% of elderly people in a tertiary care facility exhibited at least one nail change associated with aging. This is consistent with an observational study among the elderly, which revealed the presence of nail alterations in up to 98% of the population, with dull nail changes being the most encountered (71%), followed by onychorrhexis—brittleness and splitting of the nails (60%), and onychomycosis—a fungal infection of the nails (23.5%) (Rao et al., 2011). If left untreated, these nail conditions may impose a negative psychological effect and impair daily activities (Belyayeva et al., 2013). The prevalent and diverse features of nail alterations in older adults underline the significance of nails as diagnostic tools for both dermatological and systemic conditions, warranting the importance of knowledge concerning nail changes in this population.

On the other hand, the elderly group is also vulnerable to mineral malnutrition due to a high burden of non-communicable diseases, inadequate mastication, age-related gustatory disablement, reduced intake of mineral-containing foods, and poor dental health (Abeywickrama et al., 2023). Zinc—a pivotal mineral for maintaining healthy skin and nails—is often deficient in this population, leading to impairment of nail growth (Jafferany et al., 2012). Although zinc deficiency can affect individuals irrespective of age, it is more extensive in the elderly because of decreased physiological capacity. Variation in serum zinc levels is also affected by the interaction with drugs often consumed by older adults, such as antihypertensives (Braun & Rosenfeldt, 2013) and antidiabetics (Sakurai et al., 2021). In addition, accurate determination of serum zinc levels in the body is often challenging, as they are strongly influenced by serum albumin levels, which act as a zinc carrier. Furthermore, albumin levels in the elderly are often unstable, complicating the precise assessment of serum zinc levels. As a result, serum zinc may not reliably represent long-term zinc status in older adults.

In contrast, nail zinc levels reflect zinc incorporated into the nail plate during its formation over several months, rendering them less susceptible to acute albumin changes and more representative of chronic nutritional status. This renders nail zinc a more stable indicator of chronic zinc status and potentially a more appropriate biomarker for this population (Kuyumcu et al., 2013). Despite this, research on nail alterations in the elderly in relation to the nail zinc levels remains scarce. Therefore, the present study aimed to observe the potential association by evaluating the clinical and dermoscopic features of nails and measuring the nail zinc concentration in the elderly group. Understanding nail changes in the elderly population is crucial for alleviating the burden of our healthcare system precipitated by dermatological disorders, as well as aiding in the diagnosis of systemic disorders that may manifest through changes in the nails of the elderly. Moreover, identifying the factors that influence the relationship between nail zinc levels and nail abnormalities may provide insight for potential interventional strategies for managing nail changes, for instance, through zinc supplementation.

## Materials & Methods

### Ethical approval

Before the study commencement, ethical approval was sought and granted by the Health Research Ethics Committee of the Faculty of Medicine, Universitas Indonesia (reference number KET-1045/UN2.F1/ETIK/PPM.00.02/2024), with support from Dr. Cipto Mangunkusumo Hospital (letter number YR.02.01/D.IX.2.3/1000/2024).

### Study participant recruitment

This observational study employed a cross-sectional design. The minimum required sample size was calculated using the formula for comparing two independent means with a common standard deviation ($n= \frac{2{\sigma }^{2}x({Z}_{1-\alpha /2}+{Z}_{1-\beta })^{2}}{{\Delta }^{2}} $) (Rosner, 2015). Based on previous study, the standard deviation (*σ*) for nail zinc levels in the elderly population was 77.98 parts per million (ppm) (Kuyumcu et al., 2013). Assuming a two-sided α of 0.05 (Z_1−α/2_ = 1.96), 80% power (Z_1−β_ = 0.84), and a minimum detectable difference considered significant (Δ) is 55 ppm, the required sample size was estimated to be around 32 participants per group.

Subjects aged 60 years and above with or without nail alterations were invited for this study. We deployed the consecutive sampling method to the attending patients of the Dermatology and Venereology Outpatient Clinic, Dr. Cipto Mangunkusumo Hospital, Jakarta, Indonesia, from September 2024 to October 2024. Those who were on any zinc-containing supplements were excluded from the study. All eligible participants were thoroughly briefed about the study and provided written informed consent for their medical case details and accompanying clinical images to be used for publication.

### Data collection and analysis

Upon presentation to the clinic, the characteristics of study participants and their clinical and dermoscopic nail profiles were examined and recorded in a descriptive table prepared in Microsoft Excel beforehand. The diagnostic or dermoscopic criteria used for defining nail alterations in this study is described in the Supplemental File. In cases of suspected onychomycosis, potassium hydroxide (KOH)-mounted nail scrapings were performed to establish a definitive diagnosis. Afterwards, to measure the nail zinc concentration for each subject, they underwent nail clipping collection consecutively from the first to the fifth digits of fingernails and toenails until a minimal total weight of 200 milligrams was achieved. Should the necessary total nail clippings not be adequate in the first encounter, following outpatient meetings were arranged for additional collection sessions. The measurement of nail zinc levels for each subject was conducted twice in a local health laboratory in Jakarta (Laboratorium Kesehatan Daerah) by diluting the samples with HNO_3_ 65% w/w and H_2_O_2_ 32% w/w, and the inductively coupled plasma optical emission spectroscopy (ICP-AES) method (Agilent^®^ 5800; Agilent, Santa Clara, CA, USA) was harnessed. The results were reported in ppm.

Finally, relevant statistical tests were performed to investigate the association between nail zinc concentration and nail alterations, comorbidities, and medication use. Associations adjusted for potential confounders were examined using multivariable linear regression. All analyses were conducted at a significance level of 0.05 using the Stata/MP software version 19.5 for Mac. Student’s *t*-test or the Mann–Whitney U test was applied to compare the means or medians between two groups, depending on whether the data met normality assumptions. Categorical variables are presented as frequencies (percentages), while continuous variables are reported in mean ± standard deviation for normally distributed data, otherwise they are expressed in median (interquartile range).

## Results

### Characteristics of subjects

A total of 64 elderly subjects were enrolled in this study, comprising 32 case subjects with at least one manifestation in their nails and 32 controls without any nail changes. Those aged 60–69 years represented most of the study population (64.1%), while those in their 70s and above 80 years old account for 31.3% and 4.7% of the study population, respectively. Housewives and the unemployed were the most common occupations, constituting 51.6% of the total cohort. Hypertension (45.3%), diabetes mellitus type 2 (40.6%), and dyslipidemia (26.6%) were the most frequent comorbidities, while antihypertensives (42.2%), antidyslipidemic drugs (39.1%), and antidiabetics (35.9%) were the consumed medications in both case and control groups. The complete profile is delineated in Table 1.

**Table 1:** The characteristics of the study population (*n* = 64).

	**Variable**	**Case (*n* = 32)**		**Control (*n* = 32)**	
		**n**	**%**	**n**	**%**
Gender	Male	14	43.8	17	53.1
	Female	18	56.3	15	46.9
Age	60–69 years old	19	59.4	22	68.8
	70–79 years old	10	31.3	10	31.3
	≥80 years old	3	9.4	0	0.0
Occupation	Entrepreneur	4	12.5	8	25.0
	Laborer	1	3.1	3	9.4
	Civil/private employee	10	31.3	5	15.6
	Housewife/unemployed	17	53.1	16	50.0
Comorbidities^*^	Hypertension	15	46.9	14	43.8
	Diabetes mellitus type 2	12	37.5	14	43.8
	Dyslipidemia	6	18.8	11	34.4
Oral medications^*^	Antihypertensives	15	46.9	12	37.5
	Antidiabetics	13	40.6	10	31.3
	Antidyslipidemic drugs	9	28.1	16	50.0
	Anticonvulsants (for chronic pain)	4	12.5	8	25.0
	None consumed	3	9.4	8	25.0

**Notes.**

* One subject may have more than one comorbidity and consume more than one oral medication.

### Clinical and dermoscopic nail features

**Figure 1:**
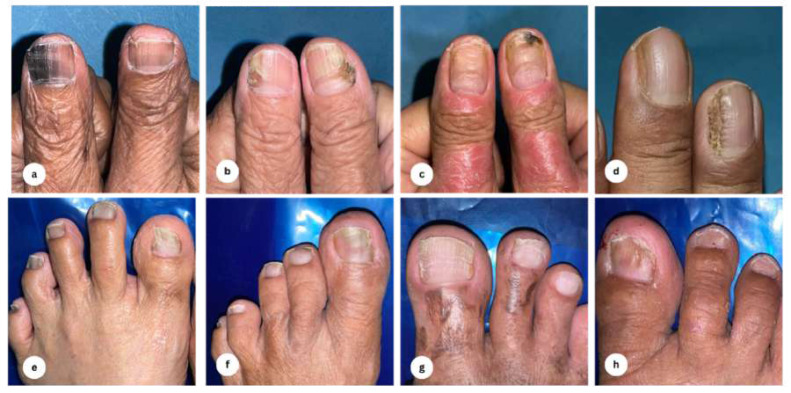
Clinical findings of nail alterations. (A) Brown-black discoloration, distal nail splitting with onychorrhexis; (B) green-brown discoloration with onychoschizia; (C) brown-black discoloration, onycholysis, and horizontal ridging; (D) clubbing finger, longitudinal ridges with lateral nail dystrophy; (E) nail dystrophy, yellow-black discoloration, onychorrhexis; (F) onycholysis, yellow discoloration, black discoloration; (G) trachyonychia; (H) spoon nail, leukonychia.

**Figure 2:**
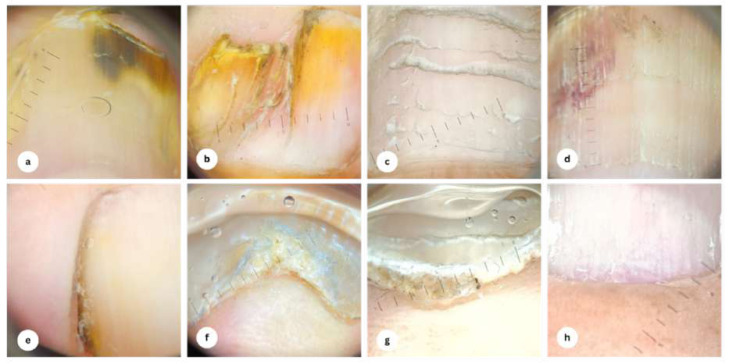
Dermoscopy findings of nail alterations. (A) Brown-black discoloration, onycholysis; (B) yellow discoloration, nail pterygium; (C) Beau’s line; (D) subungual hemorrhage, trachyonychia; (E) lateral nail fold yellow-brown discoloration; (F) onychauxis; (G) subungual hyperkeratosis; (H) splinter hemorrhage.

Anatomical distribution of the observed nail alterations among the case group is presented in Table 2. We discovered that most nail changes affected more than one digit per individual (75.0%), with the toenails being the predilection (62.3%), and the distribution was primarily unilateral for both fingernails (21.9%) and toenails (71.9%) in the elderly. As for the clinical and dermoscopic examinations of nail changes among the participants, they are demonstrated in Figs. 1 and 2, respectively. Nail plate pigmentation (*n* = 64), distal edge thickening (*n* = 39), and surface changes (*n* = 34) in both hands and feet were the most ubiquitous nail presentations in the elderly.

**Table 2:** Anatomical distribution of nail changes in the case group, presented in frequency (percentage).

**Region** ^*^	**1** ^**st**^ ** digit**	**2** ^**nd**^ ** digit**	**3** ^**rd**^ ** digit**	**4** ^**th**^ ** digit**	**5** ^**th**^ ** digit**
Right fingernails	9 (56.3%)	3 (18.8%)	2 (12.5%)	2 (12.5%)	0 (0.0%)
Left fingernails	5 (50.0%)	2 (20.0%)	2 (20.0%)	1 (10.0%)	0 (0.0%)
Right toenails	25 (71.4%)	4 (11.4%)	3 (8.6%)	3 (8.6%)	0 (0.0%)
Left toenails	21 (77.8%)	3 (11.1%)	2 (7.4%)	0 (0.0%)	1 (3.7%)

**Notes.**

* One subject may have nail changes in more than one digit.

### Nail zinc levels

**Figure 3:**
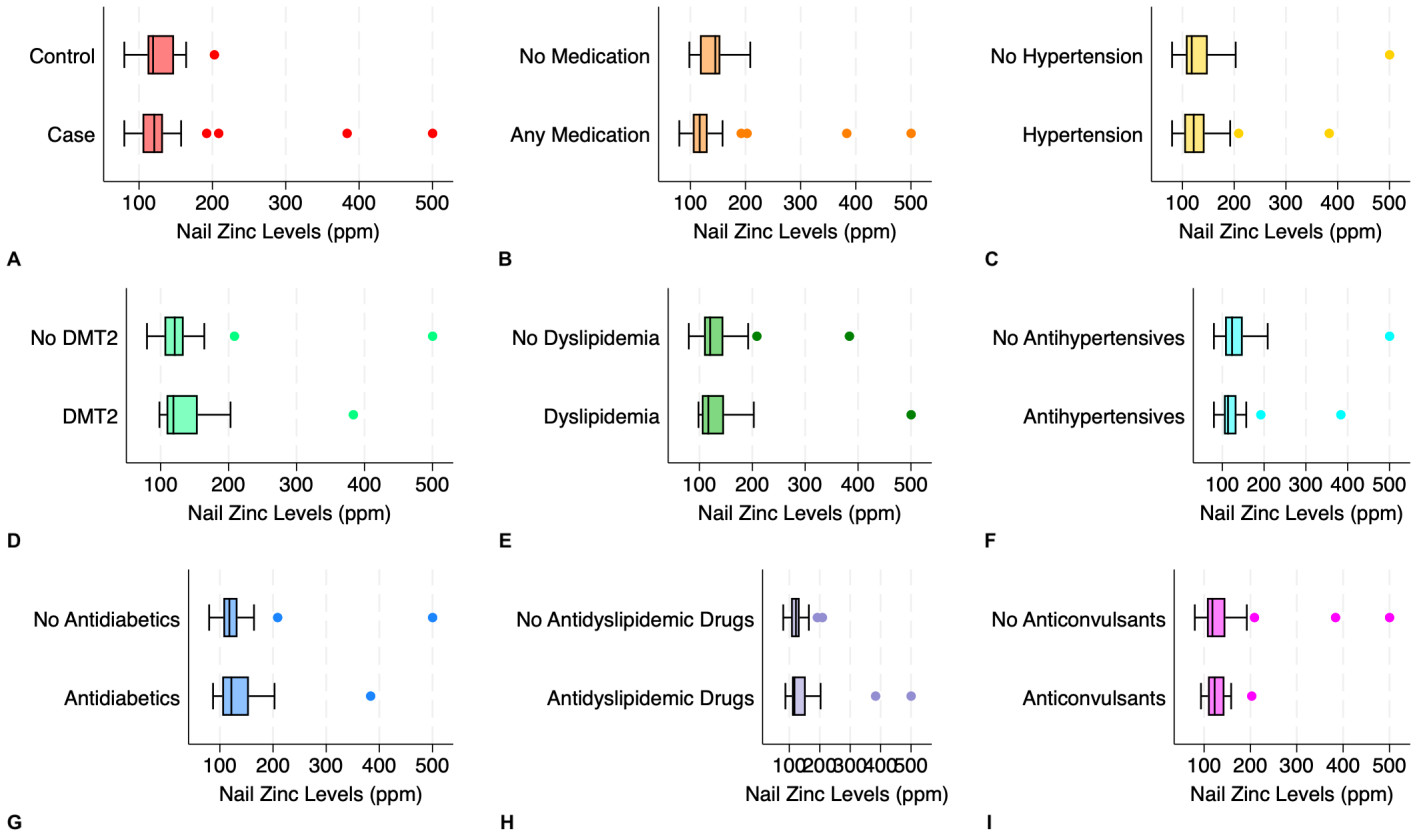
Distribution of nail zinc levels in elderly according to the presence of nail alterations, medications consumed, and comorbidities. (A) Elderly with nail changes (case) and without nail changes (control); (B) any medication *vs.* no medication; (C) hypertension *vs.* no hypertension; (D) diabetes mellitus type 2 (DMT2) *vs.* no DMT2; (E) dyslipidemia *vs.* no dyslipidemia; (F) antihypertensives *vs.* no antihypertensives; (G) antidiabetics *vs.* no antidiabetics; (H) antidyslipidemic drugs *vs.* no antidyslipidemic drugs; (I) anticonvulsants *vs.* no anticonvulsants.

The nail zinc concentrations were 120.75 (104.82–132.55) ppm among older adults with nail changes and 126.80 ± 25.81 ppm in those without nail alterations (Mann–Whitney U test, *p* = 0.687). This association remained non-significant after adjustment for comorbidities (hypertension, type 2 diabetes mellitus, and dyslipidemia) and medication use (β [95% confidence interval] = 24.46 [−9.36–58.28], *p* = 0.153). Both groups displayed a wide dispersion of nail zinc levels (79.93 to 500.30 ppm in the case group and 79.92 to 202.80 ppm in the control group), yet the mean coefficient of variation was similar between groups (5.47% in the case group and 5.31% in the control group). When grouped according to the drugs taken (any medication *vs.* no medication), the nail zinc levels were 116.94 (104.50–131.20) and 140.72 ± 30.73 ppm, respectively (Mann–Whitney U test, *p* = 0.077). Subgroup analyses based on the comorbidities and medication type revealed no statistically significant differences in nail zinc levels: (1) hypertension present *vs.* absent (121.70 (103.33–142.70) *vs.* 117.92 (106.60–148.87); Mann–Whitney U test, *p* = 0.813); (2) diabetes mellitus type 2 present *vs.* absent (118.84 (108.72–154.52) *vs.* 120.75 (105.70–133.89); Mann–Whitney U test, *p* = 0.404); (3) dyslipidemia present *vs.* absent (116.94 (104.50–146.49) *vs.* 120.34 (108.72–145.20); Mann–Whitney U test, *p* = 0.970); (4) antihypertensives *vs.* no antihypertensives (113.68 (103.94–133.89) *vs.* 123.33 (106.60–148.87); Mann–Whitney U test, *p* = 0.381); (5) antidiabetics *vs.* no antidiabetics (121.70 (104.50–154.52) *vs.* 117.92 (106.60–133.09); Mann–Whitney U test, *p* = 0.437); (6) antidyslipidemic drugs *vs.* no antidyslipidemic drugs (116.94 (108.72–153.91) *vs.* 121.70 (105.70–133.89); Mann–Whitney U test, *p* = 0.767); and (7) anticonvulsants *vs.* no anticonvulsants (128.80 ±30.73 *vs.* 118.04 (106.15–145.85); Mann–Whitney U test, *p* = 0.744). The distribution of nail zinc levels is shown in Fig. 3.

## Discussion

This study is among the first to investigate the nail alterations in older adults and their association with the nail zinc levels. We found that the first toenail is the predilection of nail changes in this population, which is consistent with previous reports showing similar findings. A plausible explanation is that, apart from the age-related degenerative nail changes, the great toenail is particularly susceptible to minor trauma from ill-fitting footwear, resulting in faulty biomechanics (Abdullah & Abbas, 2011; Albucker, Conway & Lipner, 2024). The dermoscopic features of nail plate pigmentation (melanonychia), distal edge thickening (onychauxis), and surface irregularities were also often noticed in the elderly. The discoloration of the nail plate may be due to the activation of melanocytes in the nail matrix to produce melanin in response to injury (Singal & Bisherwal, 2020). Onychauxis manifests as localized nail hypertrophy attributable to mechanical stress, such as deformities of the toes or poor footwear compatibility. Nail surface changes (for example, onychorrhexis, onychoschizia, and trachyonychia) are driven by the declining turnover rate of matrix cells in conjunction with reduced water content. They are also exacerbated by vascular insufficiency because of arteriosclerosis and histologic deterioration of dermal vessels and elastic fibers (Singh, Haneef & Uday, 2005).

In this study, we opted to utilize nail samples instead of blood or serum to measure zinc concentration, as it offers several logistic advantages, such as a minimally invasive approach, and convenient collection and storage (Gutiérrez-González et al., 2019). We found that the average of nail zinc levels of elderly individuals presenting with nail changes was slightly lower than those without, although no statistically significant variation was perceived. This is understandable since nail zinc may reflect the serum zinc levels at the time of nail synthesis, thus it is relatively stable for a long time compared to serum zinc, which fluctuates depending on physiologic processes (*e.g.*, inflammation, circadian rhythm) (Kuyumcu et al., 2013; Wessells et al., 2021). Zinc is an essential trace element required for cell structure, signalling, and as a cofactor for catalytic enzymes (Gutiérrez-González et al., 2022). The imperative role of zinc is noted in the formation of the nail plate as well, along with other micronutrients, cholesterol, and keratin (Baswan et al., 2017). Based on these facts, it is logical to deduce that the paucity of nail zinc levels in those with nail changes indicates chronic zinc deficiency (*i.e.,* approximately 3–12 months prior to nail sampling), not a recent one (Gutiérrez-González et al., 2019). Unfortunately, the cross-sectional design employed in this study limited our ability to infer causality, as the temporal sequence from the zinc deficiency exposure to the incidence of nail changes could not be established.

Subgroup analyses were also conducted to examine the possible relationship of nail zinc concentrations with the subjects’ comorbidities and medications. Similarly, we did not find marked distinctions among the tested subgroups, including hypertension, diabetes mellitus type 2, dyslipidemia, and the use of antihypertensives, antidiabetics, anticonvulsants, and antidyslipidemic drugs. Previous studies have described that oral antihypertensives (*e.g.*, angiotensin-converting enzyme inhibitors and diuretics) diminish zinc reabsorption in renal tubules, thereby increasing its excretion *via* urine and effectively lowering its concentration in plasma (Braun & Rosenfeldt, 2013; Suliburska et al., 2018). Metformin, an antidiabetic drug commonly prescribed, is thought to exert its plasma zinc-preserving effect through enhanced zinc transporters in renal tubules *via* the inhibition of hypoxia-inducible factor-1α in human kidney epithelial cells, improving zinc reabsorption (Matsui et al., 2025). In addition, patients with comorbidities (for instance, hypertension, diabetes mellitus type 2, and dyslipidemia) will theoretically have elevated inflammation and oxidative stress owing to the generated free radicals, which in turn deplete zinc levels that act as antioxidants to counter this imbalance (Ahmad et al., 2024; Ruangritchankul et al., 2023). However, in this study, we measured nail in lieu of plasma or serum zinc levels as a variable of interest, which provides stability over a prolonged period and may explain the comparability among the studied groups.

Upon closer inspection, there was a broad variation of nail zinc levels in both case and control groups. Notably, the upper range of nail zinc levels in our cohort reached approximately 500 ppm, which is considerably higher than typically reported. This wide dispersion may be attributed to biological and analytical sources of variation. Biologically, zinc levels are heavily determined by dietary intake, absorption in the gastrointestinal tract, carrier protein levels in circulation (*e.g.*, albumin, transferrin), and urinary excretion. In general, the elderly population experiences a diminution of physiological functional capacity, adversely impacting appetite and digestive efficiency, leading to malnutrition (Abeywickrama et al., 2023; Ruangritchankul et al., 2023). Serum albumin in older adults is usually decreased with the presence of malnutrition and multiple comorbidities (Cabrerizo et al., 2015), impairing the transportation of zinc systemically and into the nail matrix. Analytically, minor differences in sample preparation and intermeasurement variability may also contribute to elevated readings in a small number of samples, highlighting the need for methodological harmonization in future studies.

Although nail zinc is not affected by acute fluctuations in albumin to the same extent as serum zinc, chronically low albumin levels may still influence zinc incorporation into the nail plate during its long formation process. Since we did not measure serum albumin levels among the subjects in this study, we were unable to account for these chronic effects, which may help explain the observed variability in nail zinc levels. The implication of this variation is that establishing a universal reference range for nail zinc is challenging, as no widely accepted thresholds exist to discriminate high and low zinc values. Nevertheless, the nail zinc concentrations observed in our cohort fall within the range of previously reported values in older adults. For example, a study documented mean nail zinc levels of 123.86 ± 77.98 ppm in an elderly population (Kuyumcu et al., 2013), providing useful context for interpreting our findings despite the absence of consensus reference intervals.

This study underlines the intricate relationships among nail alterations, zinc levels, and probable confounders such as comorbidities and medications in older adults, which necessitate further exploration. We acknowledged that the lack of external control (*i.e.,* nail zinc levels of those younger than 60 years old), as compared to our study participants, may be one of the shortcomings of this study. Moreover, we did not elaborate on the nail changes, whether they were physiological or pathological, and both could concurrently happen in older adults. Pathological nail conditions, including those resulting from infections (*e.g.*, onychomycosis) or inflammatory dermatoses (*e.g.*, psoriatic nails), are highly prevalent in this population. Excluding participants with these conditions would have created a highly selected subgroup with predominantly physiological nail findings, which would not reflect real-world nail characteristics in geriatric populations. Such exclusion would also substantially reduce the number of analyzed participants, thereby diminishing statistical power and limiting our ability to detect meaningful associations. In addition, removing individuals with pathological nail changes could systematically exclude those who are more likely to exhibit both structural nail alterations and zinc imbalance, thus introducing selection bias.

For these reasons, all participants were included regardless of nail condition. Subsequent studies should incorporate an external control group, recruit larger samples to enable adequately powered subgroup analyses, clearly delineate physiological *versus* pathological nail changes, and measure serum zinc and albumin levels in addition to nail zinc levels. An observational cohort study may also be advantageous, given its superiority in establishing temporality as opposed to a cross-sectional study.

## Conclusions

From the observation, in spite of the absence of statistical significance, we discovered that the average of nail zinc levels was lower in older adults with nail changes compared to their counterparts. No substantial differences were observed when accounting for the comorbidities (hypertension, diabetes mellitus type 2, dyslipidemia) and the medication use (antihypertensives, antidiabetics, antidyslipidemic drugs, and anticonvulsants). Nevertheless, these findings justified further investigation to elucidate the complex interactions of these variables.

## Supplemental Information

10.7717/peerj.20771/supp-1 Supplemental Information 1 Raw Data

10.7717/peerj.20771/supp-2 Supplemental Information 2 STROBE Checklist for Cross-Sectional Study

10.7717/peerj.20771/supp-3 Supplemental Information 3 Diagnostic and dermoscopic criteria used for nail alterations

## Additional Information and Declarations

### Data Availability

The following information was supplied regarding data availability: As stated in Regulation Number 18 of the Ministry of Health of the Republic of Indonesia (2022), the authors are prohibited from publicly sharing individuals’ data. Anonymised data can be found in the Supplemental Information.
