# Supplementary material for: Nail structural alterations and zinc levels in the elderly: an observational cross-sectional study
Source: PeerJ. 2026 Feb 3;14:e20771. doi: 10.7717/peerj.20771 (PMC12880089; doi:10.7717/peerj.20771)
Supplement: Supplemental Information 3 [file peerj-14-20771-s003.docx]

**Supplementary Table 1**. Diagnostic and dermoscopic criteria used for nail alterations.

| **Nail part** | **Feature** |
| --- | --- |
| Nail plate | Pigmentation  Surface abnormality |
| Nail fold | Pigmentation  Vascular abnormality |
| Hyponychium | Hutchinson’s sign  Vascular abnormality |
| Distal edge | Pigmentation  Thickening  Onychoma-papilloma |
